# Contributions of smoking behaviour to lung cancer subtypes: A two-sample Mendelian randomization study

**DOI:** 10.1097/MD.0000000000051170

**Published:** 2026-10-16

**Authors:** Mohammad A. Jareebi, Ahmad Y. Alqassim

**Affiliations:** a Department of Family & Community Medicine, College of Medicine, Jazan University, Jazan, Saudi Arabia.

**Keywords:** causal inference, FinnGen, lung cancer, Mendelian randomization, smoking intensity, smoking status

## Abstract

Smoking is an established cause of lung cancer (LC), but the relative causal contributions of whether a person ever smokes and how heavily they smoke across histological subtypes remain uncertain. This study investigated the causal effects of smoking status and smoking intensity on major LC subtypes. Two-sample Mendelian randomization (MR) was performed. Genetic instruments were drawn from the GWAS and Sequencing Consortium of Alcohol and Nicotine Use: 85 variants for smoking status, a binary trait contrasting ever with never smokers, and 23 variants for smoking intensity, a continuous trait measuring cigarettes smoked per day. Outcomes were 5 LC phenotypes from FinnGen: non-small cell LC (1627 cases), adenocarcinoma (571), squamous cell carcinoma (365), small cell LC (179), and mesothelioma (133). Inverse-variance weighted regression with multiplicative random effects was the primary estimator, with weighted median, weighted mode, and MR-Egger as sensitivity analyses. Benjamini–Hochberg correction was applied across all ten tests. Analyses used R 4.2.3 with TwoSampleMR 0.7.9. Smoking intensity was causally associated with all 4 smoking-related subtypes: squamous cell carcinoma (odds ratio [OR]: 5.95 per standard deviation increase, 95% confidence interval [CI]: 3.12–11.37), small cell LC (OR: 4.43, 95% CI: 1.60–12.32), non-small cell LC (OR: 3.45, 95% CI: 2.30–5.18), and adenocarcinoma (OR: 2.66, 95% CI: 1.40–5.06). All 4 survived correction for multiple testing and were reproduced by the weighted median and weighted mode estimators. Smoking status showed positive point estimates for all 4 subtypes (OR: 1.40–1.61) but reached only nominal significance for non-small cell LC (OR: 1.51, 95% CI: 1.04–2.19, *P* = .030), which did not survive correction and was not reproduced by other estimators. Neither exposure was associated with mesothelioma. Smoking intensity shows robust causal effects on all smoking-related LC subtypes, whereas the evidence for smoking status is directionally consistent but considerably weaker. The null finding for mesothelioma, a cancer principally caused by asbestos, supports the specificity of the instruments. These results indicate that reducing consumption among established smokers warrants emphasis alongside preventing uptake.

## 1. Introduction

Lung cancer (LC) remains the leading cause of cancer mortality globally, resulting in over 1.8 million deaths worldwide in 2018.^[1]^ Although smoking is an established risk factor, the specific smoking characteristics most causally associated with different histological subtypes of LC remain uncertain. Emerging research suggests that smoking intensity and age at initiation may confer variable risks across LC types, such as adenocarcinoma and squamous cell carcinoma.^[2,3]^ Elucidating which smoking traits carry the greatest risk can focus prevention and prognostic efforts.

Observational studies have consistently demonstrated positive associations between various measures of smoking and LC risk.^[4,5]^ However, conventional observational analyses are limited by confounding and reverse causation. Mendelian randomization (MR) uses genetic variants as instrumental variables for modifiable exposures, exploiting the random allocation of alleles at conception to mimic a randomized trial.^[3]^ Because genotype is fixed at conception and cannot be influenced by disease, MR estimates are not subject to reverse causation and are less vulnerable to confounding. The approach rests on 3 assumptions: the variants must be robustly associated with the exposure, independent of confounders, and related to the outcome only through the exposure.^[6]^

MR studies have established causal effects of smoking on LC overall, but the 2 components of smoking behavior have rarely been separated. Whether a person ever starts smoking and how heavily they smoke are distinct traits with distinct genetic architectures, and they may act differently across histological subtypes.^[7,8]^ The first author has previously examined smoking behavior and health outcomes using both conventional and MR approaches^[9]^; the present analysis extends that work to histological subtypes of LC and to a direct comparison between the 2 exposures within a single analytical framework.

This study therefore compared the causal effects of smoking status and smoking intensity on 5 LC phenotypes, using genetic instruments from the largest available GWAS of smoking behavior and outcome data from FinnGen. Mesothelioma was included as a comparator: it is principally caused by asbestos exposure rather than smoking, so a null result would support the specificity of the genetic instruments rather than a generic association with cancer liability.

## 2. Methods

### 2.1. Study design

This was a two-sample MR study, in which genetic associations with the exposures and with the outcomes were obtained from separate, non-overlapping GWAS (Fig. 1). Reporting follows the STROBE-MR guideline. Because only publicly available summary-level statistics were analyzed, no individual-level data were accessed.

**Figure 1.**
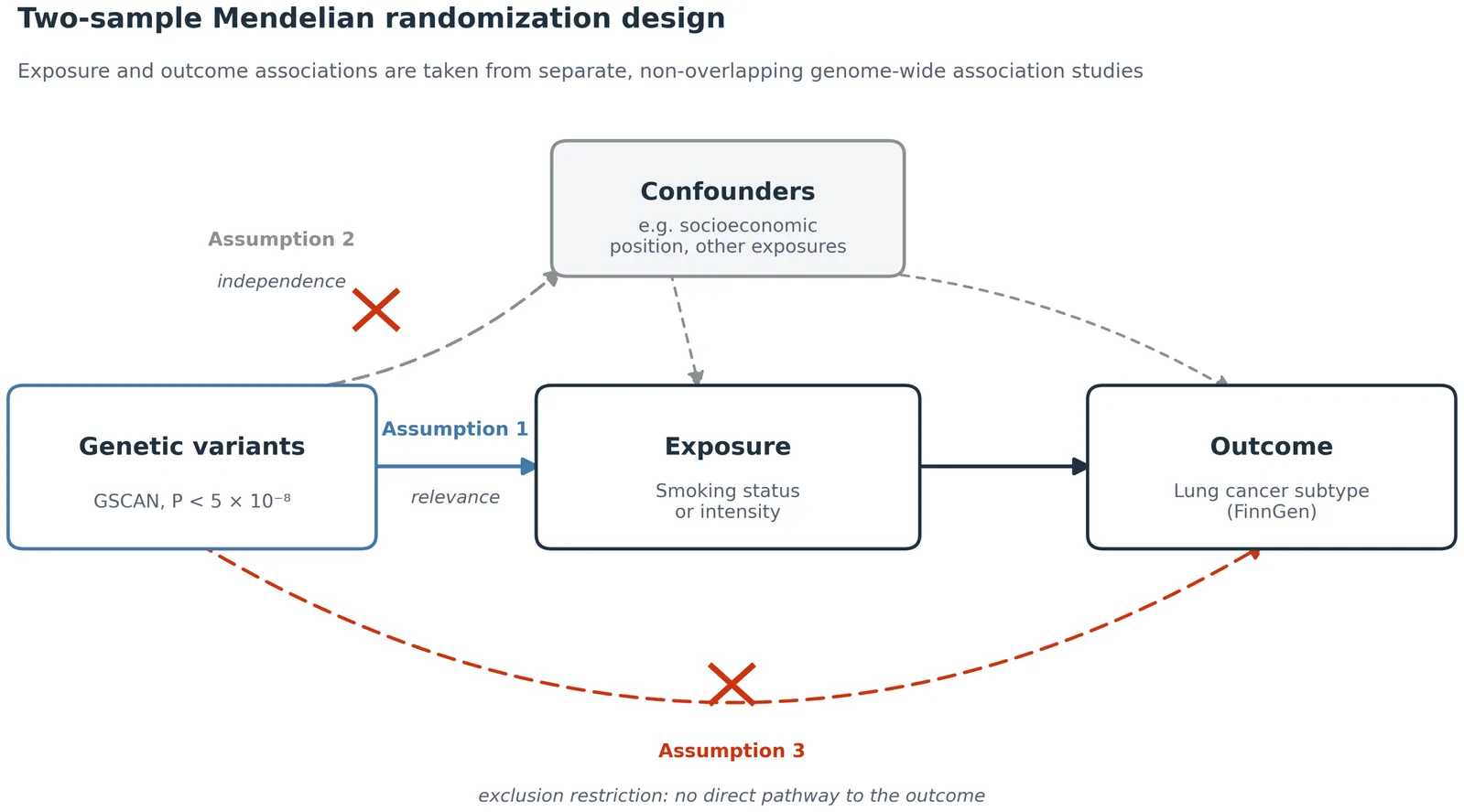
Design of the two-sample Mendelian randomization analysis and the 3 instrumental variable assumptions. Genetic variants associated with the exposure at genome-wide significance are used as instruments. Assumption 1 (relevance) requires the variants to be robustly associated with the exposure. Assumption 2 (independence) requires them to be unrelated to confounders of the exposure-outcome relationship. Assumption 3 (exclusion restriction) requires them to affect the outcome only through the exposure. Dashed lines with a cross indicate pathways that must be absent for the analysis to be valid.

### 2.2. Exposure data

Genetic instruments for both exposures were obtained from the GWAS and Sequencing Consortium of Alcohol and Nicotine Use (GSCAN), the largest available GWAS of smoking behavior.^[7]^

Smoking status was defined using the GSCAN smoking initiation trait (accession ieu-b-4877), a binary variable contrasting individuals who had ever smoked regularly with those who had never done so (n = 632,802). We use the term smoking status in preference to GSCAN’s own label, smoking initiation, because the variable records whether a person ever smoked rather than when they began; age at smoking initiation is a separate GSCAN trait, not examined here. Effect estimates are therefore expressed per unit increase in the log-odds of ever smoking, corresponding to the genetically predicted liability to ever smoke.

These are distinct constructs. Neither corresponds to age at smoking initiation, which is a separate GSCAN trait not examined here. Instruments were extracted at genome-wide significance (*P* < 5 × 10^−8^) and clumped for linkage disequilibrium (*r*^2^ < 0.001 within 10,000 kb) using the 1000 Genomes European reference panel, yielding 85 variants for smoking status and 23 for smoking intensity after harmonization.

### 2.3. Outcome data

All 5 outcomes were obtained from FinnGen, a nationwide biobank study linking genotype data to Finnish national health registers, in which cases are ascertained from hospital discharge, cause of death, and cancer registries using ICD codes.^[10]^ Using a single source ensures that all outcomes are defined consistently and that effect estimates are on a common scale, permitting direct comparison across subtypes.

The phenotypes analyzed were non-small cell LC (finn-b-C3_LUNG_NONSMALL; 1627 cases, 217,165 controls), adenocarcinoma (finn-b-C3_NSCLC_ADENO; 571 cases, 218,221 controls), squamous cell carcinoma (finn-b-C3_NSCLC_SQUAM; 365 cases, 218,427 controls), small cell LC (finn-b-C3_SCLC; 179 cases, 218,613 controls), and mesothelioma with all other cancers excluded (finn-b-C3_MESOTHELIOMA_EXALLC; 133 cases, 174,006 controls). Participants were of European ancestry. Full characteristics are given in Table 1.

**Table 1:** Characteristics of the lung cancer outcome datasets.

Outcome	GWAS accession	Cases	Controls	Total	Source	Ancestry
Non-small cell lung cancer	finn-b-C3_LUNG_NONSMALL	1627	217,165	218,792	FinnGen	European
Adenocarcinoma	finn-b-C3_NSCLC_ADENO	571	218,221	218,792	FinnGen	European
Squamous cell carcinoma	finn-b-C3_NSCLC_SQUAM	365	218,427	218,792	FinnGen	European
Small cell lung cancer	finn-b-C3_SCLC	179	218,613	218,792	FinnGen	European
Mesothelioma	finn-b-C3_MESOTHELIOMA_EXALLC	133	174,006	174,139	FinnGen	European

All outcomes were drawn from FinnGen so that effect estimates are defined consistently and are directly comparable across subtypes.

GWAS = genome-wide association study.

### 2.4. Instrument strength and harmonization

Exposure and outcome data were harmonized for effect allele alignment and strand orientation. Palindromic variants with intermediate allele frequencies and variants that could not be unambiguously aligned were excluded. Proxy variants (*r*^2^ > 0.8) were substituted where target variants were unavailable in an outcome dataset.

Instrument strength was quantified per variant as *F* = (β/SE)^2^. All instruments exceeded the conventional threshold of 10 (minimum *F* = 29.8; mean *F* = 42.1 for smoking status and 100.1 for smoking intensity), indicating that weak instrument bias is unlikely. The instruments explained 2.3% of the variance in smoking status and 3.5% in smoking intensity.

### 2.5. Statistical analysis

All analyses were performed in R version 4.2.3 using the TwoSampleMR package version 0.7.9.^[11]^ The primary estimator was inverse-variance weighted regression with multiplicative random effects. This was chosen in preference to fixed-effects inverse-variance weighted because Cochran *Q* indicated substantial heterogeneity for several exposure-outcome pairs, under which fixed-effects standard errors are understated.

Because all outcomes are binary and both exposures are modeled as continuous variables, effect estimates are odds ratios (ORs) with 95% confidence intervals (CIs) for the outcome per unit increase in the exposure. For smoking intensity this is per standard deviation increase in cigarettes per day; for smoking status it is per unit increase in the log-odds of ever smoking. This is the standard parameterization in two-sample MR and does not require the exposure to be dichotomized.

Three sensitivity analyses were performed. The weighted median estimator provides consistent estimates when at least half of the instruments are valid.^[12]^ The weighted mode estimator is consistent when the largest cluster of variants is valid.^[13]^ MR-Egger regression allows for directional pleiotropy through its intercept,^[14]^ but its slope is biased toward the null when the single nucleotide polymorphism-exposure associations are imprecisely estimated; this was quantified using the I^2^GX statistic, for which values below 0.90 indicate that MR-Egger is not informative.^[15]^ Heterogeneity was assessed using Cochran *Q* and I2.

Ten tests were performed (two exposures across 5 outcomes). Benjamini–Hochberg (BH) adjusted *P* values controlling the false discovery rate at 5% are reported alongside unadjusted values, and interpretation is based on the adjusted values.

### 2.6. Ethical approval

This study analyzed publicly available summary data. FinnGen was approved by the Coordinating Ethics Committee of the Hospital District of Helsinki and Uusimaa and complies with Finnish legislation on ethics approvals for gene-disease association studies; all participants provided written informed consent. GSCAN summary statistics derive from studies that obtained their own ethical approvals and participant consent. Because this analysis used only summary-level statistics and no individual-level data were accessed, no additional ethical approval or informed consent was required.

## 3. Results

### 3.1. Genetic instruments

After harmonization, 85 variants were available as instruments for smoking status and 23 for smoking intensity across all 5 outcomes (Table 1). All instruments were strong, with per-variant *F* statistics ranging from 29.8 to 961.0 and means of 42.1 and 100.1 respectively. The I^2^GX statistic was 0.97 for smoking intensity, indicating that MR-Egger is applicable, but 0.40 for smoking status, indicating that MR-Egger estimates for that exposure are subject to substantial regression dilution and are not informative.

### 3.2. Smoking intensity

Smoking intensity was causally associated with all 4 smoking-related LC subtypes (Table 3, Fig. 2). The strongest effect was for squamous cell carcinoma (OR: 5.95, 95% CI: 3.12–11.37, *P* < .001), followed by small cell LC (OR: 4.43, 95% CI: 1.60–12.32, *P* = .004), non-small cell lung cancer (NSCLC) (OR: 3.45, 95% CI: 2.30–5.18, *P* < .001), and adenocarcinoma (OR: 2.66, 95% CI: 1.40–5.06, *P* = .003). All 4 remained significant after BH correction.

**Table 3:** Causal effects of smoking intensity on lung cancer phenotypes.

Outcome	SNPs	IVW OR (95% CI)	*P*	BH *P*	WM OR	*P*	Mode OR	*P*	Egger OR	*P*	Egger int. *P*	*Q P*	I2 (%)
Non-small cell lung cancer	23	3.45 (2.30–5.18)	<.001	<.001	4.91	<.001	4.39	<.001	5.96	<.001	.064	<.001	57
Adenocarcinoma	23	2.66 (1.40–5.06)	.003	.010	2.93	<.001	3.36	<.001	6.06	.003	.078	.002	52
Squamous cell carcinoma	23	5.95 (3.12–11.37)	<.001	<.001	7.11	<.001	6.89	<.001	6.41	.005	.881	.119	27
Small cell lung cancer	23	4.43 (1.60–12.32)	.004	.011	4.07	.010	4.00	.008	6.81	.050	.575	.023	41
Mesothelioma	23	1.42 (0.57–3.53)	.454	.454	1.33	.651	1.54	.492	1.15	.862	.759	.643	0

Estimates are odds ratios per standard deviation increase in cigarettes smoked per day. MR-Egger is applicable for this exposure (I^2^GX = 0.97). Abbreviations as in Table 2.

**Figure 2.**
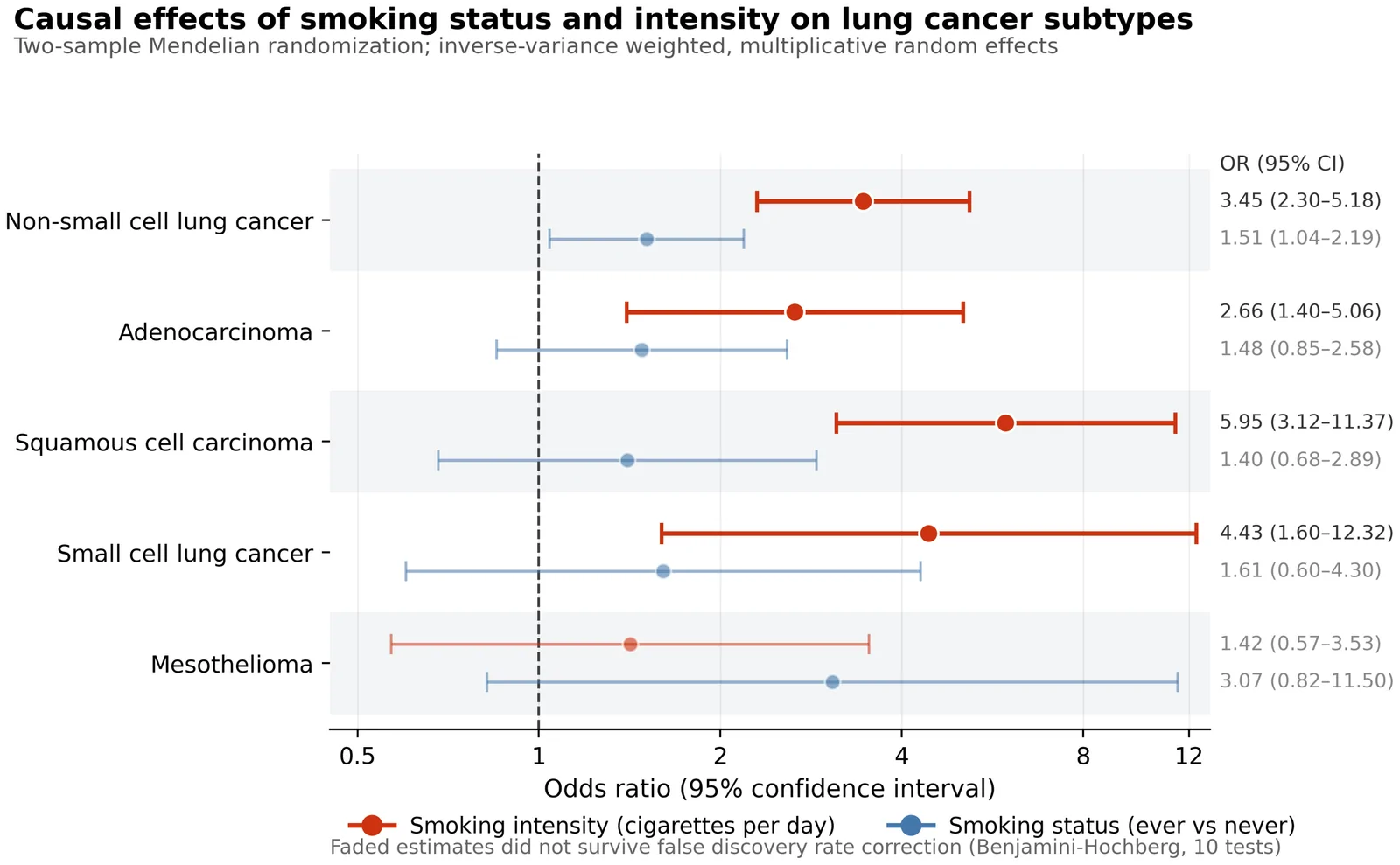
Causal effects of smoking status and smoking intensity on 5 lung cancer phenotypes. Estimates are odds ratios with 95% confidence intervals from inverse-variance weighted regression with multiplicative random effects. Smoking intensity is expressed per standard deviation increase in cigarettes smoked per day; smoking status per unit increase in the log-odds of ever smoking. Faded estimates did not survive Benjamini–Hochberg correction across the ten tests. The x-axis is on a logarithmic scale.

These associations were consistent across estimators (Table 3). The weighted median and weighted mode estimators reproduced all 4 associations at *P* < .01, with point estimates of comparable or larger magnitude. MR-Egger, which is applicable for this exposure, gave estimates between 5.96 and 6.81 across the 4 subtypes with no evidence of directional pleiotropy (all intercept *P* > .05).

### 3.3. Smoking status

Smoking status showed positive point estimates for all 4 smoking-related subtypes, ranging from OR 1.40 for squamous cell carcinoma to OR 1.61 for small cell LC (Table 2, Fig. 2). The direction of these estimates is concordant with the observational literature. However, CIs were wide, and only NSCLC reached nominal significance (OR: 1.51, 95% CI: 1.04–2.19, *P* = .030), which did not survive correction for multiple testing (adjusted *P* = .059).

**Table 2:** Causal effects of smoking status on lung cancer phenotypes.

Outcome	SNPs	IVW OR (95% CI)	*P*	BH *P*	WM OR	*P*	Mode OR	*P*	Egger OR	*P*	Egger int. *P*	*Q P*	I2 (%)
Non-small cell lung cancer	85	1.51 (1.04–2.19)	.030	.059	1.46	.138	1.28	.718	0.63	.623	.347	.054	21
Adenocarcinoma	85	1.48 (0.85–2.58)	.165	.236	1.96	.114	3.35	.255	0.45	.576	.395	.502	0
Squamous cell carcinoma	85	1.40 (0.68–2.89)	.360	.400	0.97	.957	0.40	.406	1.99	.713	.849	.264	8
Small cell lung cancer	85	1.61 (0.60–4.30)	.344	.400	1.66	.494	1.34	.862	0.13	.422	.313	.562	0
Mesothelioma	85	3.07 (0.82–11.50)	.096	.160	1.70	.552	1.66	.824	1.39	.922	.813	.022	25

Estimates are odds ratios per unit increase in the log-odds of ever smoking. The inverse-variance weighted (IVW) estimator with multiplicative random effects is the primary analysis. MR-Egger estimates are shown for completeness but are not informative for this exposure (I^2^GX = 0.40); see Methods.

BH = Benjamini–Hochberg adjusted across all 10 tests, CI = confidence interval, Mode = weighted mode, *Q* = Cochran *Q*, SNP = single nucleotide polymorphism, WM = weighted median.

This association was not reproduced by the sensitivity estimators. The weighted median gave OR 1.46 (*P* = .138), and the weighted mode OR 1.28 (*P* = .718) for NSCLC. MR-Egger estimates for this exposure were unstable, ranging from 0.13 to 1.99 with no association reaching significance, consistent with the low I^2^GX value reported above; they are shown in Table 2 for completeness but should not be interpreted as evidence for or against an effect.

### 3.4. Mesothelioma

Neither exposure was associated with mesothelioma (smoking intensity OR: 1.42, 95% CI: 0.57–3.53, *P* = .454; smoking status OR: 3.07, 95% CI: 0.82–11.50, *P* = .096; Tables 2 and 3). CIs were wide, reflecting the small number of cases (n = 133).

### 3.5. Comparison between exposures

The 2 exposures differed both in effect magnitude and in the strength of supporting evidence (Tables 2 and 3). For every smoking-related subtype the point estimate for smoking intensity exceeded that for smoking status, by factors ranging from 1.8 for adenocarcinoma to 4.2 for squamous cell carcinoma. More importantly, all 4 associations with smoking intensity survived correction for multiple testing and were replicated across 3 independent estimators, whereas no association with smoking status survived correction or was replicated by any sensitivity analysis.

### 3.6. Heterogeneity and pleiotropy

Cochran *Q* indicated significant heterogeneity for 4 of the ten analyses (smoking intensity with NSCLC, adenocarcinoma, and small cell LC; smoking status with mesothelioma; Tables 2 and 3), which motivated the use of multiplicative random effects throughout. The MR-Egger intercept did not differ significantly from zero for any analysis (all *P* > .05), providing no evidence of directional pleiotropy, although this test has limited power for smoking status given its I^2^GX value.

## 4. Discussion

This two-sample MR analysis found that smoking intensity has robust causal effects on all 4 smoking-related LC subtypes examined, while the evidence for smoking status is directionally consistent but substantially weaker. Neither exposure was associated with mesothelioma.

### 4.1. Relation to previous work

This is not the first MR study of smoking and LC. Previous analyses have established a causal effect of smoking on LC overall,^[7,8]^ and the first author has previously examined smoking behavior in relation to a range of health outcomes using both conventional and MR methods.^[9]^ The contribution of the present study is threefold: it separates the 2 components of smoking behavior within a single framework, it examines histological subtypes rather than LC as a composite, and it draws all outcomes from a single biobank so that estimates are directly comparable across subtypes.

The magnitude and ordering of the effects for smoking intensity align with the observational literature. Case-control and cohort studies have consistently reported the strongest associations between smoking quantity and squamous cell carcinoma and small cell LC, with weaker associations for adenocarcinoma.^[2,16,17]^ The present estimates reproduce that ordering: squamous cell carcinoma 5.95, small cell 4.43, and adenocarcinoma 2.66. That an approach with entirely different sources of bias recovers the same pattern strengthens the case that this ordering is causal rather than an artifact of recall or confounding.

The weaker evidence for smoking status requires cautious interpretation. Observational studies report clear associations between ever smoking and LC, so the absence of a robust genetic signal here is more plausibly a matter of statistical power than of biology. The instrument for smoking status explains 2.3% of exposure variance, and with between 133 and 1627 cases per subtype, the analysis has limited power to detect effects of the magnitude estimated. It is also relevant that smoking status captures liability to start smoking without reference to how much or how long a person subsequently smokes, so its total effect on LC is expected to be diluted relative to a direct measure of consumption.

### 4.2. Biological interpretation

The more pronounced effects of smoking intensity may be explained by greater exposure to carcinogenic compounds from higher cigarette consumption. Smoking more cigarettes daily delivers larger doses of carcinogens, including polycyclic aromatic hydrocarbons, tobacco-specific nitrosamines, and volatile organic compounds, which induce DNA damage and promote malignancy.^[18]^ The particularly strong effect on squamous cell carcinoma is consistent with its typical origin in the central bronchial airways, which receive the highest carcinogen deposition, whereas adenocarcinoma arises more peripherally and shows the weakest association here.^[2]^ Smoking-induced DNA methylation changes provide a further plausible mechanism linking cumulative exposure to carcinogenesis.^[19]^

### 4.3. Mesothelioma as a comparator

Mesothelioma is principally caused by asbestos exposure, and although smoking interacts with asbestos in LC, it is not an established independent cause of mesothelioma. The null findings for both exposures are therefore the expected result and serve as a form of negative control: they indicate that the genetic instruments are not simply capturing a generic liability to cancer or to socioeconomic disadvantage, which would have produced associations across all outcomes. This interpretation should be made with the caveat that the mesothelioma analysis included only 133 cases and had correspondingly wide CIs.

### 4.4. Strengths and limitations

The strengths of this study include the use of genetic instruments to reduce confounding and exclude reverse causation, strong instruments for both exposures, outcomes drawn from a single biobank on a common scale, examination of 4 estimators with differing assumptions, correction for multiple testing, and the inclusion of mesothelioma as a comparator.

Several limitations should be acknowledged. First, both cohorts are of European ancestry, which limits generalizability to other populations in whom smoking patterns and LC incidence differ. Second, the analysis is underpowered for the rarer subtypes, particularly small cell LC and mesothelioma, and the null findings for smoking status should not be read as evidence of no effect. Third, MR-Egger was not informative for smoking status because of regression dilution, so pleiotropy is less thoroughly excluded for that exposure than for smoking intensity. Fourth, smoking status and intensity are genetically correlated, and univariable MR estimates their total rather than direct effects; multivariable MR would be required to separate these and is a natural extension of this work. Finally, the exposures capture lifetime genetic liability rather than smoking at any particular age, so these estimates should not be read as the effect of an intervention delivered at a specific point in life.

## 5. Conclusion

This two-sample MR study provides genetic evidence that smoking intensity is causally associated with all 4 smoking-related LC subtypes examined, with effects that survive correction for multiple testing and are consistent across estimators. The evidence for smoking status is directionally concordant with observational findings but considerably weaker, and no association survived correction. The null result for mesothelioma supports the specificity of the instruments.

These findings indicate that the quantity smoked, not merely whether a person smokes at all, is a principal determinant of LC risk, and that the effect differs across histological subtypes in a pattern consistent with differential carcinogen deposition in the airway. From a public health perspective, they support giving weight to reducing consumption among established smokers alongside continued efforts to prevent uptake.

## Acknowledgments

The authors thank the participants and investigators of FinnGen and of the GWAS and Sequencing Consortium of Alcohol and Nicotine Use, whose publicly available summary data made this analysis possible.

## Author contributions

**Conceptualization:** Mohammad A. Jareebi, Ahmad Y. Alqassim.

**Data curation:** Mohammad A. Jareebi.

**Formal analysis:** Mohammad A. Jareebi.

**Investigation:** Mohammad A. Jareebi.

**Methodology:** Mohammad A. Jareebi, Ahmad Y. Alqassim.

**Project administration:** Ahmad Y. Alqassim.

**Resources:** Ahmad Y. Alqassim.

**Supervision:** Ahmad Y. Alqassim.

**Writing – original draft:** Mohammad A. Jareebi.

**Writing – review & editing:** Mohammad A. Jareebi, Ahmad Y. Alqassim.
